# Research Ethics with Gender and Sexually Diverse Persons

**DOI:** 10.3390/ijerph17186615

**Published:** 2020-09-11

**Authors:** Mark Henrickson, Sulaimon Giwa, Trish Hafford-Letchfield, Christine Cocker, Nick J. Mulé, Jason Schaub, Alexandre Baril

**Affiliations:** 1 School of Social Work, Massey University, Auckland 0745, New Zealand; 2 School of Social Work, Memorial University of Newfoundland, St. John’s, NL A1C 5S7, Canada; sgiwa@mun.ca; 3 School of Social Work and Social Policy, University of Strathclyde, Glasgow G4 OLT, UK; trish.hafford-letchfield@strath.ac.uk; 4 School of Social Work, University of East Anglia, Norwich NR4 7TJ, UK; Christine.Cocker@uea.ac.uk; 5 School of Gender, Sexuality & Women’s Studies, York University, Toronto, ON M3J 1P3, Canada; nickmule@yorku.ca; 6 Department of Social Work and Social Care, Birmingham University, Birmingham B15 2TT, UK; j.schaub@bham.ac.uk; 7 École de Service Social/School of Social Work, University of Ottawa, Ottawa, ON K1N 6N5, Canada; abaril@uottawa.ca

**Keywords:** bisexual, gay, gender diverse, human ethics committees, lesbian, research ethics, transgender, ethical principles

## Abstract

Identifying and developing inclusive policy and practice responses to health and social inequities in gender and sexually diverse persons require inclusive research ethics and methods in order to develop sound data. This article articulates 12 ethical principles for researchers undertaking gender and sexually diverse social, health, and related research. We have called these the ‘Montréal Ethical Principles for Inclusive Research.’ While writing from an international social work perspective, our aim is to promote ethical research that benefits people being researched by all disciplines. This paper targets four groups of interest: 1. Cisgender and heterosexual researchers; 2. Researchers who research ‘general’ populations; 3. and sexually diverse researchers; 4. Human ethics committees. This article was stimulated by the 2018 Global Social Work Statement of Ethical Principles, which positions human dignity at its core. It is critically important to understand and account for the intersectionality of gender and sexuality with discourses of race, ethnicity, colonialism, dis/ability, age, etc. Taking this intersectionality into consideration, this article draws on scholarship that underpins ethical principles developed for other minoritized communities, to ensure that research addresses the autonomy of these participants at every stage. Research that positions inclusive research ethics at its foundation can provide a solid basis for policy and practice responses to health and social inequities in gender and sexually diverse persons.

## 1. Introduction

Over the past half-century, gender and sexually diverse persons have increasingly emerged as a legitimate focus in research (by gender and sexually diverse persons we mean everyone who identifies as part of the lesbian, gay, bisexual, queer, intersex, trans, and nonbinary communities, however they identify themselves). The early 1980s was a pivotal time for research, as the global HIV epidemic focused researchers’ attention on gay men, and eventually men who have sex with men; on lesbians through women’s rights; and trans people. However, as social, political, and cultural attitudes evolve, this attention has broadened to include people identifying as gender and sexually diverse and has attracted the attention of researchers in multiple contexts with varying agendas. This is particularly true for researchers who are interested in health and social inequities for gender and sexually diverse persons around the world (we use the word ‘inequities’ in this paper, because it is inequities, or the lack of justice, that lead to inequalities in health and social outcomes). For the most part, this is a positive development, because research has the potential to validate the existence of these communities and to highlight the rich complexities within these populations. While the findings that emerge from such research are important for planning services in areas such as health, mental health, and social care, this is not their only purpose: such findings can also help planners, policy makers, service providers, and theorists to understand behavior, constructions of identity, and the ways knowledge itself is understood and validated. For policy planners and intervention designers who seek to address health inequities that lead to inequalities, having good evidence to develop their responses is essential. However, some social work theorists [1,2] remind us that exclusively focusing research on gender and sexually diverse communities draws our attention away from stigmatizing and oppressive heteronormative and cisgendered environments and suggests that people fit easily into discreet and discernible categories. As a result, it is important to retain a critical focus on cisnormative and heteronormative discourses and the nefarious effects of essentializing people for ease of research planning. (Baril [3] (pp. 94–95) defined cisnormativity as the normative component of the cisgenderist system, an oppressive system made by and for cisgender people (i.e., non-trans people) discriminating against trans people. Ansara [4] (p. 15) defined cisgenderism as follows: ‘Unlike “transphobia”, which emphasizes individual hostility and negative attitudes, the cisgenderism framework incorporates both unintentional and well-intentioned practices. Cisgenderism often functions at systemic and structural levels: even when individuals might reject some aspects of cisgenderist ideology, they may live and work within broader structural contexts that perpetuate and manufacture cisgenderism.’).

This paper is intended to contribute to the debate about ethical issues raised by research associated with gender and sexually diverse communities. It is not our intent to set out methodological guidelines on how to do research with gender and sexually diverse communities, but rather to suggest some ways to address the ethical challenges raised by this work. There have been a number of calls to make research with marginalized populations more representative and to address the autonomy of participants; this paper is situated within this wider movement. These appeals have called for active and appropriate engagement with various marginalized populations at all stages of the research process, from conceptualization to dissemination [5,6,7]. These various contributions have foregrounded the debates explored in this paper, as a way to address the concern that knowledge and power is being gained from the exploitative study of minority groups [3,8,9]. This paper re-affirms the importance of meaningfully engaging with the communities being studied.

It is not our intent to revisit widely accepted ethical norms and standards of social research [10], but rather to interrogate their heterocisnormativity, and through that examination, extend those norms and standards as they relate to gender and sexually diverse individuals, communities, and researchers. While there has been some attention paid to the need for increased research in gender and sexually diverse communities, especially in the area of health and mental health [11], there has been more limited work on the ethics of research in this area, although there have been some recent proposals of ethical principles with trans and non-binary participants [12,13]. We seek to address this lacuna and introduce some possible ways to address the challenges of research with gender and sexually diverse people. While we write from a social work context, the focus of this paper is a more general audience of social and health researchers, specifically four groups:

- Cisgender and heterosexual researchers doing research with gender and sexually diverse persons and communities;
- Researchers who research ‘general’ populations, which will inevitably include gender and sexually diverse persons;
- Gender and sexually diverse researchers doing research with gender and sexually diverse persons and communities;
- Human ethics committees (as they are known in local and national contexts) that are responsible for reviewing research proposals and ensuring that the proposals meet the expected ethical standards.

The impetus for this paper is the ratification of the Global Social Work Statement of Ethical Principles (GSWSEP) by the general bodies of both the International Association of Schools of Social Work and the International Federation of Social Work in July 2018. Elaborating the GSWSEP is beyond the scope of this paper, and readers can familiarize themselves with the context and background of these principles and the commentary if they are not already [14,15]. While the GSWSEP ethical principles are social work-specific, they are useful here because, together with other commentators, they have generated a discussion about the lack of broader social research ethics with gender and sexually diverse individuals and communities [12,13,16]. This discussion, in turn, led the authors to develop these principles. At the core of GSWSEP is ‘Recognition of the inherent dignity of humanity’ (Principle 1), which suggests that individuals are “Far from being autonomous and independent beings as constructed by liberal theory, as human beings we are all embedded in societies and dependent on their socio-political, economic and cultural structures and conventions” (p. 1). The principle of dignity implicitly encourages social researchers to focus both on gender and sexually diverse communities and on the oppressive and binarized heterocisnormative environments in which those lives are lived.

## 2. Background

Gender and sexually diverse communities comprise multifarious persons who experience themselves as radically and subjectively different from cisgender heterosexual majorities. This difference is frequently experienced as hidden, ignored, stigmatized, or devalued. While gender is a more commonly shared experience, the ways gender is enacted differs considerably across cultures. Many people, including academic researchers, uncritically assume an essentialized and conflated understanding of sex as assigned at birth as correct and enduring. For trans, gender fluid, and intersex persons [17], gender may be misassigned at birth, and may change over the life course (or even day-to-day) or may simply not conform to so-called traditional biological or cultural gender binaries of women and men. The notion of sexuality as identity is one that has emerged from liberal (as opposed to relational, or ‘collectivist’) cultures, which allow and even prioritize individualized identities [18,19]. Other rights movements have also required essentialized taxonomy in order to secure identity-based rights [20]. The increasing application of categorical language such as gay, lesbian, bisexual, and so forth, has ended up defining persons, rather than persons refining the categories. Nevertheless, diverse sexualities (and often genders) have been expressed and even honored throughout history in many cultures [21,22,23,24,25,26,27,28,29,30,31,32]. We acknowledge that liberal humanist, or ‘Western,’ discourses and identities are not translatable across all cultures, and these discourses do not readily accommodate some cultures with highly diverse understandings of gender and sexuality [33]. Some countries and cultures have professional, ethical, and legal codes that are at odds with each other when it comes to issues of gender and sexual diversity [34]. This conflict not only makes the researcher vulnerable, but more importantly it requires individuals in these communities to make difficult choices to either participate in research or access services. It is helpful to heed the writing about those experiences from members of those communities [35] and build on scholarship [7,13,36] to encourage social researchers in all disciplines to consider their practices.

Too often researchers ignore lived realities: that for some people gender is misassigned or mutable, and that sexual identity comprises not only behavior and desire, but the array of various sexual story possibilities told in many cultures [37,38]. Intersectionality, a theoretical framework that emerged from Black feminist writers, refers to the complex ways different aspects of identity and oppression work simultaneously to shape individuals’ lived experiences [39], and can allow for these lived realities to become known [40]. However, intersecting identities and oppressions such as gender, sexuality, race, ethnicity, culture, caste, and class can be overlooked when undertaking data analysis. The dominant positivist assumption posits that these components can be studied in isolation. Research on the wider population rarely considers the array of gender and sexuality differences and their overlap with other social identities, which may be included in research populations. In addition, gender and sexually diverse populations may be at significant personal, social, or political risk if their identities become known in cultures or states where their identities or activities are socially stigmatized or criminalized; as a result, they may collaborate with researchers in concealing themselves, or not challenge researcher assumptions and stereotypes [41,42,43]. Therefore, these circumstances are likely to mean that important stories are not told, and that the research is incomplete. As demonstrated by other scholars, ethical research will include protection for participants (for instance, by changing any identifying details) so that their stories can be told in their entirety, thereby improving the findings and their impact.

We recognize that the taxonomy of identities discussed here is contested and often fraught, and that language is also dynamic. In this paper, we use the terms ‘gender and sexually diverse’ as the most inclusive language (for now) because they acknowledge that both gender and sexuality occur on spectrums. We contrast this with the terms ‘gay,’ ‘lesbian,’ ‘bisexual,’ etc. because these are based on liberal notions of static categories [33], prioritize these identities at the expense of other identities and social roles [44], and may not apply in non-Western and indigenous cultures. Some populations defy traditional categorizations, such as the Two-Spirit Indigenous peoples of North America, who fulfill third gender cultural roles [45].

While we challenge formulaic categories of identity, for some people these categories remain important and are used in popular discourse and the media. However, some communities, which lack culture-specific terminology for identifying gender or sexual diversities, may default to these categories, meaning they are reproduced in incongruous contexts. The word ‘queer’ has been reclaimed by many, but by no means all, gender and sexually diverse persons from its historically hateful use and redeployed in an empowering way. For some people, the term ‘queer’ also signifies their personal celebration of difference and how this difference contributes to diversity, as opposed to a mainstream, assimilationist agenda. We also hold, with UNAIDS [46], that persons should not be reduced to initials or acronyms (e.g., ‘LGBT&c’) even for editorial convenience. We acknowledge that this can result in some awkward and even repetitive linguistic constructions.

## 3. Reviewing Relevant Debates

Over the last several decades, progress has been made in response to challenges for doing research in gender and sexually diverse communities. These include Meezan and Martin [47], who explored the challenges of applying traditional ethical notions to research with identified gender and sexually diverse communities. Research, however, on gender and sexually diverse communities has, in some contexts, been met with opposition from human ethics committees. This may be because it has a political, rather than a scientific, purpose [48]; does not fit with state-enforced social values [34]; occurs in contexts where human rights are perceived as hegemonic liberal humanist discourse; or occurs where gender and sexually diverse persons are perceived as a threat to the political or social regime [49,50]. It is hard to imagine how ethical research on gender and sexually diverse persons can exist in cultures where human rights themselves are not respected, even as we recognize the limitations of human rights [51,52]. All research on gender and sexually diverse persons emerging from oppressive contexts must be received and interpreted with extreme caution. That is not to say that researchers should merely accept the status quo, but in these contexts, researchers will want to ally carefully with on-the-ground community-based organizations in order to ensure that their research both meets the needs of local gender and sexually diverse communities and does not put them further at risk. Brown writes: “The very existence of a universal declaration [of human rights] rebukes long-standing, but intellectually feeble presumptions, that a sovereign state’s treatment of its citizens is the business of that state and that state alone” [53] (p. 2). We suggest that this is also of interest to researchers, because increased human mobility, interdependencies of nation states, and ongoing changes in treaties, policies, and law challenge fixed notions of sovereignty [54].

It is important to reflect and debate the existing scholarship on ethical principles with other minority or marginalized groups of people, to move ahead with these challenging issues [55]. Research inclusivity with individuals and communities is important, but brings challenges [7]. Using broader ethical frameworks is recommended by some authors, with the suggestion that broader frameworks assist researchers to align themselves with the needs of the communities they research [56]. There are also concerns raised about the links between research and the oppression of minority individuals, with the situation for trans people highlighted by Marshall et al. [16,57]. These approaches have presented various guidelines that range from a mapped protocol [16] to setting out six areas of discussion [13] and nine guidelines [12].

Social researchers are often grouped into ‘insiders’ and ‘outsiders’ when describing connection to the community under study. There are both advantages and challenges to either position, but there is no space here to examine these in detail. While an insider may have a quicker and more intense understanding of context [58], such a status brings challenges that include confidentiality and boundaries, as some of these communities are highly interconnected. Insider research can also be marginalized by other researchers [3,8] who claim that insider research includes an ‘agenda’ (with the presupposition that outsider research is agenda-free). A significant issue with outsider research is the risk of universalizing experiences, suggesting that one individual’s experience represents all others from the same group. However, outsider research can have significant advantages of funding, reputation, and networks.

It is likely that researchers will want to understand each community and sub-community on its own terms. For instance, bisexuality is not an in-between identity, but an entirely different identity; bisexual persons can be made invisible by being lumped together with other groups, with key differences ignored by cisgender, heterosexual, and queer researchers [59]. Understanding communities on their own terms and in all their complexities becomes even more important when considering intersectional identities. For example, a trans adolescent new migrant still living with their birth family must negotiate multiple and competing roles and identities [60]. The young participant’s life is a lived reality, and the researcher’s instruments, experience, and epistemic framework should assist the participant to engage positively with the research encounter.

Particular issues have been identified in research with gender and sexually diverse young people, and especially young people who are runaway or throwaway, who have cognitive or physical differences, or who have mental health or substance misuse issues [61]. Human ethics panels may express concern about young people participating in research without parental or guardian consent, yet obtaining such consent to participate in research as a gender or sexually diverse young person may put the young person at significant risk [16,62,63,64]. Importantly, young people do not require parent or guardian consent to experience themselves as different. Requiring guardian consent effectively silences the voices of gender and sexually diverse young people, and we are reminded that “[t]he principle of respect for persons demands protection of those more vulnerable, not exclusion” [65] (p. 629). We propose that one way of reframing these challenges may be for research ethics panels to focus more on the rights of the young person to be heard than on the rights of the parents to give permission [63,66]. Researchers have found that young people from the age of 14 are capable of making adult-level decisions to participate in research when the information is provided in language appropriate to their age [62]. There is related case law in the United Kingdom to support the Gillick competencies on the rights of children to make medical decisions, and the Fraser guidelines on the provision of contraceptive information [67]. This is particularly important, as gender and sexually diverse youth will often have had sexual initiation and more partners than cisgender and heterosexual youth Eaton et al., cited in [62].

## 4. Principles for Ethical Research with Gender and Sexually Diverse Persons and Communities

It is with this background, context, and theoretical foundations that we propose the following principles for researchers and ethics committees, as one contribution to defining more explicit principles for ethical research with gender and sexually diverse communities. We propose that they be identified as the Montréal Ethical Principles for Inclusive Research, after the city where the authors first developed the concept for this paper. We acknowledge that social issues (and our responses to them) are constantly changing, and that gender and sexually diverse persons and communities are dynamic. Setting out explicit best practice guidelines, therefore, would become quickly outdated. It is our hope that researchers can reflect on and develop research designs and proposals with more considered and sensitive practices that are responsive to these principles. We also acknowledge that in some contexts these principles may be aspirational; however, by proposing them, we support researchers who wish to be accountable to their participants but may be prevented from doing so in full by the practical realities of their institutions, ethics panels, funders, or budgets.

### 4.1. Respect the Dignity of All Research Participants

This is a foundational principle in GSWSEP [14], from which most of the other principles here elaborate. Naturally, dignity is experienced differently by different people, and it is important that participant experiences of dignity prevail over researcher notions. Respecting dignity can be as simple as routinely using the pronoun used by the participant about themselves, and as complex as ensuring that participants or consultants are appropriately compensated for their time and expertise (although we recognize the fraught debates around compensation in research, which go beyond the scope of this paper). Respecting dignity means meaningful consultation from the initial planning stages of a research project, through data collection, data analysis, conclusions, recommendations, and dissemination of results. Respecting dignity avoids ‘othering’ language in findings. Whether or not the researcher is a member of these communities, we encourage them to ask questions from a position of openness and humility. Researchers should seek to learn from the lived experiences of their participants; participants are the experts in their lives. If their experiences differ from what the researcher expects, this provides an opportunity for learning and expanding knowledge. Research questions can be drawn from the communities of interest so that they are relevant, respectful, and interesting to participants. Researchers should consider whether they are excavating knowledge for the benefits of researchers, or for public use with little direct value to the community. If participants seem difficult to engage, or do not offer much information, it may be that the study is not interesting or relevant to them, or the researcher’s position does not appear sufficiently open. If the researcher is not a member of these communities, it may be helpful to request a community member to provide an introduction.

### 4.2. Engage with the Taxonomy and Language of Participants

Taxonomy includes self-reference, categories, pronouns, and all other vocabulary and terminology, regardless of how transient, localized, or ‘unscientific’ such terms may seem. Researchers should be encouraged to consider and use the taxonomy of the ways these various communities identify themselves, rather than requiring that they fit into pre-existing categories on a form. While this may make the data analysis more complicated and more time-consuming, and therefore, possibly more costly, the right to self-identify is one of the most fundamental of rights. Using the language of the participant community communicates respect for their right to self-determination and respects their lives. Language provides insight and using (and explaining) the language that people use dignifies them and will enrich the research.

### 4.3. Examine Assumptions about Who is and is not in the Sample Population

We recommend that researchers assume that gender or sexually diverse persons or groups will be in any sample, regardless of how participants are selected. Research not focused on this group should not assume, for instance, that research participants are all cisgender or heterosexual, or not bisexual or intersex. Researchers will want to consider whether they have established ways for gender and sexually diverse persons to disclose themselves and participate fully in the research. While categorized identities are convenient for data entry, they do not always suit individuals’ identities. To encourage accurate and meaningful data collection, we suggest questions can be framed to assist participants to participate meaningfully. If, for instance, sex options are ‘male’ or ‘female’ only, how are trans or intersex persons meant to answer that question (and it may be helpful to consider whether sex is a required variable in all studies)? Adding ‘other’ to these options is literally to ‘other’ participants. One option would be to ask members of gender and sexually diverse communities to read through any questionnaire, survey, or interview schedule before it is administered to ensure that language and response options are as inclusive as they possibly can be [7,55].

### 4.4. Assume that Binarized Cisgender Heteronormativity will have an Impact on the Lived Experiences of Gender and Sexually Diverse Research Participants

We recommend researchers develop a plan to address or manage the impact of assuming binarized cisgender heteronormativity, including protecting participant identities and data. If the researcher is working in a context, for instance, where same-sex sexuality is criminalized or stigmatized, then participants will be at best reluctant to disclose themselves to the researcher. In some places, this will mean that participants live a concealed identity because of the very real danger of arrest, torture, involuntary surgery or other involuntary medical or ‘treatment’ intervention (such as so-called reparative therapy), or even execution, and these threats should be considered carefully. In other contexts, there may be threats of public humiliation, stigmatization, loss of employment, accommodation, child custody, family connections, or other social benefit or status. Simply participating in research, waiting for an interview, or meeting with researchers in public may represent a very real threat to participants. Living minority stress [68] may also lead to other equally pernicious but less severe consequences, such as self-stigma, self-censorship, isolation, and psychological sequelae such as anxiety, depression, or suicidal ideation. It is important to remember that gender and sexually diverse participants may participate in projects but may conceal important and potentially significant aspects of their experiences, thus resulting in a kind of heterocisnormative reporting bias.

### 4.5. Recognize Intersectionality and Its Impact, Including Indigeneity, Race, Ethnicity, Religion, Class, Gender, Age, Language, Culture, Colonization, Dis/Ability, and More

Intersectionality is not an additive analysis of social categories of identity, where sexual orientation, for example, is added on to race or vice-versa. This approach to conceptualizing multiple dimensions of identity implies that categories of oppression are mutually exclusive and independent of each other, such that one form of oppression or discrimination does not influence the other. An intersectional approach, however, acknowledges the indivisible and interdependent nature of social categories of identities and oppressions, and works from the premise that forms of privilege and disadvantages contribute to and maintain structures of domination [69,70]. Thus, we encourage researchers to consider the implications of intersectionality in the design, data analysis, and dissemination of the project findings. In the design and data analysis stages, researchers may consider which methods and procedures would best enable the complexity of participants’ experiences to be known, so that their voices could be centered in a more holistic and natural way. For example, in a research study looking at the use of pre-exposure prophylaxis (PrEP) among Black men who have sex with men, an intersectional framework would suggest that researchers move beyond a biomedical approach to HIV prevention, to consider some of the intersecting and structural barriers to PrEP uptake, such as difficulty paying for PrEP, stigma related to PrEP use, and a lack of access to or poor health care provider contact due to racism. Such an approach assumes an integrated approach to knowledge creation, in which relevant participants and stakeholders are engaged from the start through to the application of research evidence. The knowledge coproduced can then be used to drive equitable policies and community solutions tailored to the needs of affected community members, across intersecting identities and experiences. As a result, qualitative or mixed-methods research may be more valuable for capturing complex experiences of intersectional stigma and oppression, which cannot be easily isolated in purely quantitative studies.

### 4.6. Acknowledge Multiple Epistemologies

The nature of gender and sexual diversity is that each person comes to their own understandings about self and others through their own experience. Researcher questions will always mean different things to different people depending on their personal experiences and contexts. However, it is important to remember that a gender or sexual minority’s way of knowing (including intersectional experiences within) is different from a cisgender heterosexual way of knowing [71]. A gender and sexually diverse way of knowing and validating truth is radically subjective and also relies on disclosure. Disclosure may be verbal (declarations, or even confrontations) or non-verbal (clothing, gender presentation, buttons and badges, etc.).

### 4.7. Appreciate that Information from Gender and Sexually Diverse Persons and Communities Acts Indigenously

This means, firstly, considering pre-existing meanings on research data. Establishing meaningful reference or consultation groups, or including cultural advisors, are ways to ensure that researcher interpretations are respectful and sympathetic to the ways they were intended, and accurate in meaning. Ensuring that individuals represented in the research are involved in the design and dissemination of the research is a way of returning data and findings to the communities from which they were gathered [36]. It is important that this participation, either as members of the research team (or as advisor or collaborators) should avoid tokenism [6]. While such inclusion is challenging given the increased research costs and time, individuals can often feel either tokenized or experience research fatigue if inclusion is not done well.

### 4.8. Avoid Problematizing or Pathologizing the Lived Experiences of Gender and Sexually Diverse Research Participants

Resilience and resourcefulness should be recognized alongside difficulties, problems, and challenges [72]. Gender and sexually diverse persons will usually be surprisingly resilient because they have navigated stigma, minority stress, and microaggressions [73] all their lives [4,74]. Diversity has long been constructed as a problem of diverse communities, rather than of dominant communities, and as Fish [1] and Hicks [2] have written, it is the exclusiveness of dominant heterocisnormative cultures that should be problematized. Research plays an important role in advancing the interests of individuals, groups, communities, and societies, and not merely identifying problems. We recommend that researchers are mindful of research fatigue in over-researched communities and populations. A priority of ethical research is to ensure that the most pressing issues for individuals and communities are considered, and that findings are appropriately disseminated to participating communities as well as to decision-makers.

### 4.9. Interrogate Researcher (or Ethics Panel Member) Assumptions and Experiences (Whether or Not the Researcher or Panel Member is an Insider or Outsider to the Community)

It is important for researchers and ethics panel members to be reflective about taken-for-granted cis- and heteronormative assumptions as well as insider assumptions about participants. If the researcher understands themselves as a member of a gender or sexual diverse community, we propose that it is still incumbent on them to avoid cis-, trans-, and homonormativity. There are many ways to live these experiences, especially across national boundaries or cultural spaces. An insider role is confined to one’s own community and experiences and will not reflect the entire diversity of experiences of any group. This is an important challenge, because studies about the lived experiences of indigenous and racialized gender and sexually diverse and trans groups continue to be produced by white gay men. If the researcher is not a member of these communities, they will want to give serious consideration to how best they can reflect the experiences of the communities they are researching [12].

### 4.10. If a Participant is (Legally) a Young Person or Other Dependent Person, Prioritize the Informed and Voluntary Consent of the Research Participant Over the Need for the Consent of a Guardian

This issue has been explored above and has been set out by a number of researchers. Human ethics panels and researchers can be anxious about young people participating in social research without parental or guardian consent, yet obtaining such consent to participate in research may put the young person at significant risk for violence and other negative consequences at home. At the same time, excluding them from social research is to silence them and restrict their contributions. Researchers could consider how to present study information in an age-appropriate way and to ensure that the young person understands the nature of the project and that their participation is completely voluntary [12]. When these conditions are met, then guardian consents are not necessary. Human ethics panels should examine these concerns and incorporate them into their ethics standards and procedures.

### 4.11. Ensure Adequate Compensation for the Time Participants Commit to the Research Project

We recognize that this is a difficult area and this principle can be a challenge for both researchers and funders. Nevertheless, participants are experts in their own lives, and it is important that this expertise and participant time is recognized fairly. An essential element of research budgets is appropriate compensation to participants for their time. Similarly, if consultation or cultural advisors are used, their contribution should be appropriately compensated. We suggest that adequate compensation of participant and advisor time and expertise should be considered standard, not an addendum. In the case of indigenous and racialized gender and sexually diverse and trans people, current research strategies often struggle to consider the deepening racialization of poverty experienced by people whose stories we want to document [13]. We are not suggesting that research funds should be paid to lift participants out of poverty—it cannot. However, researchers and funders can aspire to do better to compensate people who are the only experts in their own lives for the effort required to tell their stories, which can be difficult and (re)traumatizing. What constitutes adequate compensation is an important topic for discussion with individuals, groups, and communities at the center of the research project and the institutions of researchers. Part of ethical research may include challenging funding structures that contribute to these problems in order to strengthen research and make research more responsible.

### 4.12. Generate Theory from the Lives of Research Participants

Generating theory from the lives of research participants is especially important in the case of indigenous and racialized gender and sexually diverse peoples. Wherever possible in developing foundations of a project or in interpreting findings, reference should be made to works by gender and sexually diverse authors, and especially those authors who identify as members of racial and cultural groups that are not white. In this way, communities define and shape their own knowledges, in ways that accord with their sense of being and place in the world. Knowledge production takes place in a contested social, political, cultural, and economic context. Centering the knowledges of racialized and otherwise minoritized and marginalized gender and sexually diverse people can affirm their lives, histories, and subjugated standpoints. These standpoints are usually different from dominant knowledge practices, on account of their epistemological resistance to ongoing colonial narratives of racial/ethnic and cultural inferiority, which works to silence the voices of marginalized populations. Situating subjugated knowledges at the center of research permits alternative accounts of theories about the social world, where dominant theories are not uncritically assumed to speak for all people [6,12].

## 5. Conclusions

Developing this paper across national, cultural, and linguistic boundaries has been a challenging experience. All the authors of this paper understand themselves as gender or sexually diverse persons as well as experienced researchers who bring an intersectional lens to research with gender and sexually diverse peoples. Each of us has brought with us the cultural, national, social, and political contexts and norms that inform our work and our writing. These contexts varied, among other matters, even in the use of capitalization, vocabulary, and language. While these things may appear minor at first glance, they nevertheless reflected the much larger and complex realities and discourses in which we live and work. Through self-reflection and respectful discourse—and a certain amount of accommodation—we were able to come to shared understandings of what we offer as basic principles of research ethics with gender and sexually diverse persons. We have observed racist, colonial, cisgenderist, and heteronormative research that has marginalized, made vulnerable, or excluded the experiences of gender and sexually diverse persons—in other words, poor research.

To counteract this, our paper offers a contribution to what we have reflected upon and consider as minimum principles of good research in gender and sexually diverse communities. We are mindful, of course, that one paper cannot meet all needs, and local contexts will require further development and elaboration. We are aware that in many contexts these principles may begin as aspirations. We want to support those aspirations to become realities, particularly within our own research and discipline. Our nuanced articulation of principles of respect for human dignity and consultations with local communities encourage locality-enriched research. As we have noted above, communities and language are dynamic and evolving, and today’s “edgy” language can become tomorrow’s oppressive cliché. Again, we offer these principles to encourage researchers to be reflective and consultative, to assist them to meet dynamic communities and identities with dynamic research processes. It is important to examine the problem of using an ethical template to measure whether a research proposal is inclusive and respectful of gender and sexually diverse persons. Rather, we suggest that all people engaged in the research enterprise can make an ongoing commitment to research that is inclusive, dynamic, and responsive to evolving language, communities, and expressions of gender and sexual diversity. In this way, researchers can provide sound evidence on which to base policy and interventions to address health and social inequities for all persons, and particularly for gender and sexually diverse persons.
